# Breast cancer screening in women at increased risk according to different family histories: an update of the Modena Study Group experience

**DOI:** 10.1186/1471-2407-6-210

**Published:** 2006-08-17

**Authors:** Laura Cortesi, Daniela Turchetti, Isabella Marchi, Antonella Fracca, Barbara Canossi, Battista Rachele, Ruscelli Silvia, Pecchi Anna Rita, Torricelli Pietro, Federico Massimo

**Affiliations:** 1 Centro per lo Studio dei tumori familiari, Dipartimento di Oncologia ed Ematologia, Università di Modena e Reggio Emilia, Italy; 2 Registro Tumori di Modena, Italy; 3 Dipartimento di Diagnostica per Immagini, Università degli Studi di Modena e Reggio Emilia, Italy

## Abstract

**Background:**

Breast cancer (BC) detection in women with a genetic susceptibility or strong family history is considered mandatory compared with BC screening in the general population. However, screening modalities depend on the level of risk. Here we present an update of our screening programs based on risk classification.

**Methods:**

We defined different risk categories and surveillance strategies to identify early BC in 1325 healthy women recruited by the Modena Study Group for familial breast and ovarian cancer. Four BC risk categories included BRCA1/2 carriers, increased, intermediate, and slightly increased risk. Women who developed BC from January 1, 1994, through December 31, 2005 (N = 44) were compared with the number of expected cases matched for age and period. BRCA1/2 carriers were identified by mutational analysis. Other risk groups were defined by different levels of family history for breast or ovarian cancer (OC). The standardized incidence ratio (SIR) was used to evaluate the observed and expected ratio among groups. All statistical tests were two-sided.

**Results:**

After a median follow-up of 55 months, there was a statistically significant difference between observed and expected incidence [SIR = 4.9; 95% confidence interval (CI) = 1.6 to 7.6; p < 0.001]. The incidence observed among BRCA carriers (SIR = 20.3; 95% CI = 3.1 to 83.9; *P *< 0.001), women at increased (SIR = 4.5; 95% CI = 1.5 to 8.3; *P *< 0.001) or intermediate risk (SIR = 7.0, 95% CI = 2.0 to 17.1; *P *= 0.0018) was higher than expected, while the difference between observed and expected among women at slightly increased risk was not statistically significant (SIR = 2.4, 95% CI = 0.9 to 8.3; *P *= .74).

**Conclusion:**

The rate of cancers detected in women at high risk according to BRCA status or strong family history, as defined according to our operational criteria, was significantly higher than expected in an age-matched general population. However, we failed to identify a greater incidence of BC in the slightly increased risk group. These results support the effectiveness of the proposed program to identify and monitor individuals at high risk, whereas prospective trials are needed for women belonging to families with sporadic BC or OC.

## Background

Following the discovery the mutant BRCA1 and BRCA2 genes which predispose carriers for BC and OC [1,2], many high-risk women request genetic testing and counselling on strategies to reduce their risk of death from BC. There are several options available for the management and care of women at risk for developing BC. Primary prevention can be achieved by prophylactic mastectomy and/or other risk-reducing strategies, e.g. chemoprevention with Tamoxifen, or oophorectomy. Prophylactic mastectomy is usually not proposed as the first choice for the management of women at high risk for developing BC, although it has been demonstrated to be efficient in reducing the incidence of BC either in women belonging to families with familial [3] or hereditary BC [4,5]. Otherwise, secondary prevention through intensified surveillance to identify the earliest possible diagnosis of familial BC at a prognostically favourable stage is considered a feasible and acceptable strategy. If intensified surveillance is chosen, we have to keep in mind that there is difference between women with a positive genetic test who face a lifetime risk of 46–87% [6,7] and women with a calculated lifetime risk of ≥ 18%, according to specific models, e.g. Claus [8,9]or Gail [10]. Depending on BRCA status or family history, management of women at increased risk for developing breast cancer has to be considered as an individual surveillance program. The most widely recommended strategy for BRCA carriers entails frequent, intense surveillance that begins at age 25 to 35 years. How to screen these patients is also debatable. There is no consensus on the optimum modality and screening interval of women identified to be at moderate or high risk of breast cancer. [11,12]. We know that the "lead time" will be short, in BRCA related BC, due to this forms rapid growth rate, and the screening performed at shorter intervals [13]. Furthermore, although mammography remains the gold standard tecnique, the radiation risk, due to the fact that BRCA genes are implicated in DNA repair of double stranded breaks typically caused by ionizing radiation [14,15], and the diminished sensitivity in dense breasts, lead to evaluate the efficacy and safety of other imaging screening modalities for breast cancer, such as ultrasound and MRI. In particular, ultrasound has an established role in complementing diagnostic mammography in young patients with dense breasts [16-18]. Considering MRI, two retrospective [19,20] and five prospective studies [21-25] have suggested that this imaging technique is useful in screening high risk women.

Several guidelines for hereditary breast cancer (HBC) and familial BC have been published. Particularly, for familial breast cancer, three groups of risk have been defined (high, moderate and low), according to the number of affected relatives, the age at onset and the cancers associated. All women belonging to moderate/high risk should be offered annual mammography, but MRI and ultrasound should not be used in routine surveillance [26,27]. With the objective of improving the impact of different BC screening strategies in women in different risk categories, we developed operational criteria for the selection of family groups at risk of developing BC. Four categories were defined and four different BC screening strategies were established, in terms of age to begin screening, screening intervals, and imaging techniques. Here we describe the results of our surveillance and document the effectiveness of the proposed program in selecting individuals at risk.

## Methods

### Study population

Beginning in 1994, 1628 family histories were collected by the Modena Study Group for Familial Breast and Ovarian Cancer, in accordance with an oncologist-based model of cancer genetic counselling for HBC and hereditary OC (HOC) [28]. The family histories were obtained through detailed questionnaires and interviews. Family pedigrees were traced as far backward and laterally as possible, including a minimum of four generations and extended to paternal lines. The BC risk estimate was assessed according to the Gail model [10], Claus tables [8,9] and a slightly modified BRCAPro model, adapted to the Italian population as suggested by Marroni et al. [29]. Furthermore, risk estimation was also determined according to the following criteria: 1) at least three relatives diagnosed with BC or OC in two different generations; 2) at least one of the three relatives must be a first-degree relative of one of the other two; in the case of male interposition, a relationship of different degree is allowed; 3) at least one BC must be diagnosed before the age of 40 years or be bilateral; 4) at least one BC diagnosed at age ≤ 35, regardless of family history; 5) at least one BC and one OC in the same woman, regardless of family history; 6) at least one male BC, regardless of family history; 7) one sporadic BC or OC. Applying these criteria, subjects were classified at high, intermediate, or slightly increased risk as described in Table 1. We defined the lifetime risk cut-off, calculated by the Gail model, at 30–50% in the high risk group, 18–29% in the intermediate risk group, and 6–18% in the slightly increased risk group. Finally, a group defined by a genetic predisposition due to the presence of the mutant BRCA genes was established to have a lifetime 50–85% risk of developing BC.

After disclosure of the BC risk to the consultants, they were instructed to convey suggestions concerning surveillance to relatives who were at an increased risk with respect to the general population.

To date, 1325 asymptomatic women accepted to be enrolled in our surveillance program, of which 1072 were first degree, and 253 were second degree relatives; 48 women belonged to carrier group (37 were BRCA1 mutation carriers, and 11 BRCA2 mutation carriers), 674 belonged to the high risk group, 257 to the intermediate risk group, and 346 to the slightly increased risk group (Table 2).

Additionally, 299 healthy women belonging to collected pedigrees did not adhere to our surveillance program for the following reasons: a) consultants failed to communicate the information to other relatives due to a poor relationship between family member; b) although they were informed of belonging to an at-risk family, they decided not to undergo surveillance due to low interest or because they lived too far from the clinic.

### Genetic testing

Genetic testing to identify deleterious BRCA1 and BRCA2 mutations included direct automated sequencing on the entire coding sequence. From 1995 to 2005, 385 index cases affected by BC or OC, in the high risk categories, were tested for BRCA1/2 mutations of which, 82 carrier patients (21.3%) were identified. Of these 82 carriers, 79 asymptomatic relatives were found to carry a BRCA mutation.

### Surveillance

An open prospective nonrandomized study was designed and approved by the Ethical Committee of Modena. Carriers of BRCA1 or BRCA2 mutations discovered through genetic testing or subjects at risk according to our previously described criteria, who were at least 18 years of age were eligible. Women with symptoms that were suggestive of BC or women who had a personal history of BC were excluded.

From January 1994 to September 2000, surveillance consisted of mammography (oblique and craniocaudal views and, if necessary, compression views and magnifications), ultrasonography and clinical breast examination (CBE) for BC prevention, and transvaginal ultrasound plus Ca.125 serum levels for early diagnosis of OC were proposed at different intervals based on the assessed risk. In October 2000, surveillance for carriers of BRCA1 or BRCA2 mutations was modified by introducing a dynamic breast MRI with gadolinium-containing contrast medium (Table 3). Whenever possible, the 4 independent exams for each screening event were planned on the same day during the second week of the menstrual cycle in premenopausal women. When indicated, additional investigation with fine-needle aspiration or core biopsy was performed.

### Data collection and statistics

All data regarding family and individual characteristics, surveillance program, follow-up, additional investigations, and the final outcome of each examination from the years 1992 to 2005 were collected in a database. A person-year approach was used to evaluate the BC incidence. Person-years of risk were calculated from the baseline visit to the BC diagnosis (at surveillance or in the interval between two examinations) or for those without diagnosis, to the end of the study period (December 31, 2005). Detection rates were expressed as the number of events per 1,000 person-years of follow-up, and confidence intervals (CI) were calculated using the exact method [30]. Expected cancer incidence for consultants and for all first-degree and second-degree relatives in the lineage at risk older than 18 years was based on age-specific Modena Cancer Registry (MCR) rates from 1998 through 2002 in 5-year age groups, beginning at age 25 years and ending with age 85 years or older [31]. The observed women-years at risk were then multiplied by expected cancer incidence obtained from the MCR database to estimate the total number of cancers expected. Standardized incidence ratios (SIRs) were determined by calculating the ratio of observed to expected numbers of cancers. An "interval cancer" was defined as any cancer presenting between two regular screening rounds. Interval cancer rate was defined as the number of women with a diagnosis of an interval cancer per 1,000 person-years at risk. Sensitivity of the screening test was calculated as the ratio of BCs detected by surveillance divided by the total number of BC (screen-detected plus interval cancers). The chi-square test was used to calculate *P *values. All statistical tests were two-sided.

## Results

### Clinical characteristics

**Figure 1:**
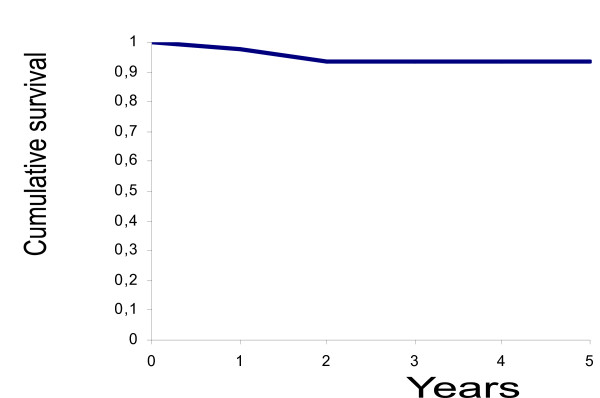
*Actuarial five year survival rate*. Actuarial five year survival rate was 93%. After a median follow-up of 55 months four recurrences and three deaths were observed, two for disease progression and one for heart failure.

After a median follow-up of 55 months (range 1 to 151 months), a total of 44 breast tumors including 28 infiltrating (64%) and 16 ductal carcinomas in situ (DCIS) (36%) were found in women belonging to different risk categories. Five cancers occurred in BRCA carriers (4 infiltrating and 1 DCIS), 23 in the high risk group (14 infiltrating, 9 DCIS), 11 in the intermediate risk group (8 infiltrating and 3 DCIS), and 5 in the slightly increased risk group (2 infiltrating and 3 DCIS). Among the 28 invasive tumors, 21 were ductal, 6 lobular, and one was a tubular carcinoma. All patients were surgically treated. Of the 44 patients, 30 (68%) received conservative surgery, 14 (32%) had a mastectomy of which 6 were bilateral. At the time of diagnosis, 17 patients were diagnosed with stage I (63%), 7 with stage II, 2 with stage III, and 2 with stage IV. Eight (29%) tumors were less then 10 mm in diameter, 10 (36%) were between 10 and 15 mm, 9 (32%) were greater than 15 mm (range 16 to 50 mm) and one was an inflammatory BC. Ten (36%) were node-positive. Five patients were treated with hormonal therapy, 8 patients received chemotherapy, 11 patients were treated with chemotherapy and hormonal therapy, and 7 received surgery only. In 8 cases, chemotherapy was anthracycline-based. After a median follow-up of 55 months, 4 recurrences and 3 deaths were observed, 2 for disease progression and one due to heart failure. The actuarial 5 year survival rate was 93% (Fig. 1).

### Efficacy of screening

With a total number of follow-up years of 6,066, the BC detection rate (invasive and in situ BC) was 7.3 per 1000 person-years and 4.6 per 1000 person-years excluding DCIS. Of the 44 patients who developed BC, 11 (25%) were palpable and 33 (75%) were nonpalpable tumors. Thirty-six of the 44 tumors were detected at screening (5 during the first round and 31 at a subsequent round) making the rate of screen-detected cancers 5.9 per 1000. Eight cancers, all identified by CBE, were diagnosed in the interval between screening events (interval cancer rate 1.3 per 1000). The diagnosis was made only by CBE in 4 cases, (one inflammatory carcinoma, 2 nipple bleeding, and one axillary metastasis), by CBE plus ultrasonography in 3 cases, and by CBE plus ultrasound plus mammography in one case. The time interval from the last negative screen until diagnosis ranged from 1 to 14 months. The characteristics of patients with interval cancers are shown in Table 4.

Among the 36 screen-detected BC, 3 were palpable and 33 were nonpalpable cancers. Twenty-eight were diagnosed with mammography (78%), 18 with ultrasound (50%), 35 with mammography plus ultrasound (97%), and 4 by MRI (100%) (Table 5). An MRI was performed only in BRCA carriers and one BC was detected only by this imaging technique.

Eight DCIS were detected in women aged less than 50 years and 8 in women older than or equal to 50 years. The screening sensitivity increased with age with a low rate in the age group <50 (65%) and a very high rate (93%) in the oldest age group, with an overall sensitivity of 82%.

The detection rates of BC were 31.6 per 1000 person-years in BRCA1/2 gene mutation carriers, 6.9 per 1000 in the high risk-group, 9.9 per 1000 person-years in the intermediate risk group, and 3.5 per 1000 in the slightly increased risk-group. The incidence of BC in the entire study population was significantly higher than expected (SIR = 4.9; 95% confidence interval (CI) = 1.6 to 7.6; p < 0.001). The incidence was significantly higher among BRCA1 and BRCA2 carriers (SIR = 20.3; 95% CI = 3.1 to 83.9; *P *< 0.001) and amongst subjects classified at high (SIR = 4.5; 95% CI = 1.5 to 8.3; *P *< 0.001) or intermediate risk (SIR = 7.0, 95% CI = 2.0 to 17.1; *P *= 0.0018). However the incidence of BC was not higher than expected in the group classified at slightly increased risk (SIR = 2.4, 95% CI = 0.9 to 8.3; *P *= 0.74) (Table 6). Finally the SIR of BC was 14.4 (95% CI 6.7–26.5, *P *< 0.001) in the group of women aged less than 50 years and 3.3 (95% CI 1.9–5.5 *P *< 0.001) among women aged 50 years or older.

## Discussion

Our clinical and radiological imaging surveillance program led to the detection of 44 breast cancers, including 28 infiltrating and 16 in situ. The SIR for BC was very high overall for the women in the study (4.9, *P *< 0.001) and BRCA carriers (20.3, *P *< 0.001) compared to that expected in the general age-matched population. In the high and intermediate risk groups the SIR reached statistical significance (4.5, *P *< 0.001 and 7.0, *P *= 0.0018, respectively) confirming the effectiveness of our current approach in the identification of women at increased risk. On the other hand, a low SIR (2.4, *P *= 0.76) was detected in the slightly increased risk group. Interestingly, a much higher proportion of DCIS (36%) was detected in our screening compared with patients from an age-matched population not considered at increased risk (9%). Furthermore, the performance of our screening compares favourably with the recommendations of the European Commission for quality assurance in mammography screening [32] which indicates a good detection rate in the first round where it was more than 3.5 fold the incidence rate before screening (2.6‰) and more than 1.5 fold in the subsequent rounds. The combination of mammographic and ultrasound screening in women with a family history was further investigated. As in other studies, where ultrasound was useful for bridging the relatively long time interval between the annual surveillance rounds [33,34], adding ultrasound to mammography improved the sensitivity of screening from 78% (28/36) to 97% (35/36). In all age groups and risk categories, ultrasound showed a very high sensitivity in addition to mammography with the exception of the slightly increased risk group where no cancer was detected by this modality. As expected, the major advantage of ultrasound was seen in women aged less than 50 years where the sensitivity was up to 100% (from 7 to 11 of 11 cancers). Finally, although performed in a limited number of cases, breast MRI screening showed high sensitivity in women with a genetic predisposition for BC. In fact, an interval cancer in a BRCA carrier patient, had already appeared on an MRI, but was considered an intramammary lymph node. A retrospective evaluation of the preceding mammograms was performed for all interval cancers, with the exception of one 32 year old woman (Table 4) in the slightly increased risk group who had never had a mammography before diagnosis. An interval cancer in a 51 year old woman at high risk was considered a missed cancer because the previous mammogram identified a mammary sprain at the left upper outer quadrant, although a fine needle aspiration was negative for atypical cells (Table 4). Also a DCIS in a 45 year old woman at intermediate risk must be considered a missed cancer, since the patient had a nipple discharge that was negative for atypical cells. Excluding these cases, the real detection rate of interval cancer was 0.8 per 1,000. All the invasive interval cancers, except for 1 DCIS, had a tumor grading of 3. Nevertheless, the 5 years disease-free survival and overall survival for interval cancers was 100%, suggesting the high compliance of women followed at our institution. The overall percentage of tumors with positive lymph nodes was 36% (10 of 28) with no difference in age or risk group. The mean number of lymph nodes removed was 24. Other studies have reported a lower 10 – 35% node positivity. The higher rate in our study may be due to different patient population characteristics, such as age or screening schemes and modalities, or to our more stringent node sampling.

The Saetersdal study [35] reported a detection rate of 15 per 1,000 on 537 women at risk for BC, who were selected on the basis of autosomal dominant inheritance with DCIS accounting for 11% of all cancers. Kollias [36] performed a screening on 1371 women less than 50 years old with a family history of BC; 23 invasive cancers were detected during a mean follow-up of 22 months. The incidence for invasive breast cancer was 7.9 per 1000 women-years, with a SIR of 5 when compared with an age-matched female population in the U.K. Six carcinomas in situ (21%) were detected, suggesting that young women at risk of BC due to family history may benefit from regular breast screening for the early detection of in situ lesions. Lalloo [37] selected 1259 women under the age of 50 with a positive family history and a lifetime risk of BC of 1 in 6 or greater. In this population, 12 cancers were detected giving a SIR of 1.42, 95% CI 0.73–2.48. The percent of node-positive tumors (45%) was very high. Chart [38] identified 24 tumors (invasive and in situ) in 1044 women distributed in three categories (high, moderate, and slightly increased risk). All screen-detected tumors were in situ or stage I, suggesting that surveillance of women at increased risk for breast cancer may be useful in detecting disease at an early stage. Finally, Brekelmans [39], who has enrolled 1198 women characterized by BRCA1/2 mutations or by a BC risk over 15% between 21–70 years of age in a screening program, found 35 cancers (31 invasive and 4 DCIS) after a median follow-up of 3 years. The SIR for invasive cancers was 7. Furthermore he had a 74% screening sensitivity.

Both the Kollias and Lalloo studies analyzed young women aged less than 50 years. In our study, 897 women were under 50 years of age and 306 were older. The SIR of BC in women aged <50 years was higher than that observed in the U.K. screening program (14.4 vs.5), while this ratio decreased in women aged more than 50 years.

The significance of detecting DCIS in mass screening programs is unknown. It is estimated that the risk of invasive cancer following untreated DCIS in the general population is 30–50% and this usually occurs within 10 years. In the context of a family history, several investigators believe detection of these non-invasive lesions may become more important. The meaning of lobular carcinoma in situ (LCIS) is debatable. This lesion was recently considered as a high risk premalignant lesion such as atypical ductal hyperplasia, papillomatosis, and so on [40].

In conclusion the detection rate and the observed versus expected ratio found in our surveillance program were in accordance with the risk group, with a statistically significant value for BRCA carriers, and for the high and intermediate risk groups. Also, the screening sensitivity was improved in the above groups, but fell in the slightly increased risk group. In this latter group, no significant differences in detection rates and SIR were found with respect to the expected number of BCs based on age-specific Modena Cancer Registry (MCR) rates from 1998 through 2002. As already reported by other authors [41], early breast cancer screening does not seem to be cost-effective in women belonging to a slightly increased risk group. Newer imaging technologies, such as MRI, may offer a better technique for the early diagnosis of breast cancer, especially in BRCA1/2 gene carriers. Furthermore, with the objective of reducing the number of interval cancers, randomized trials should be designed utilizing MRI in the high and intermediate risk groups, as already proposed by other authors [23,33].

Finally, our operational criteria seemed effective in identifying people at increased risk of developing breast cancer, and are currently being evaluated in a larger group of individuals from families followed at Institutions of the Italian Network on Hereditary Breast and Ovarian Cancer.

## Conclusion

In conclusion, our data show that the proposed screening program was able to select individuals at risk, in agreement with the Italian Network on Hereditary Breast and Ovarian Cancer. This paper provides evidence based proof that an appropriate surveillance program can identify a relevant number of breast cancers at an early stage in a population at risk.

## Competing interests

The author(s) declare that they have no competing interests.

## Authors' contributions

LC participated in the design of the study and drafted the manuscript.

DT participated in the design of the study.

IM acquired the data of the center

AF acquired the data of the Modena Cancer Center and performed the statistical analysis.

BC performed the surveillance screening.

RB performed the surveillance screening

SR carried on the genetic counselling

AP participated the MRI study.

PT coordinated the radiological screening

MF designed the study and revised the final manuscript.

All authors read and approved the final manuscript

## Pre-publication history

The pre-publication history for this paper can be accessed here:



## Figures and Tables

**Table 1:** Modena model

**High risk**			Pedigree classification	
I) at least 3 relatives diagnosed with BC (or OC) in 2 different generations	II) one BC/OC case is a first-degree relative of the other 2 (of the other 1 if the first criterion is not fulfilled)°	III) at least one case has been diagnosed at the age ≤ 40 or with bilateral BC		
•	•	•	***H*** *ereditary*	HBC/*HBOC
•	•		***S*** *uspected ****H*** *ereditary*	SHBC/SHBOC
•		•	***S*** *uspected ****H*** *ereditary*	SHBC/SHBOC
BC diagnosed at age ≤ 35, regardless of family history			***E**arly **O**nset*	EOBC
BC and OC in the same woman, regardless of family history			***B**reast **O**varian **C**ancer*	BOC

**Intermediate risk**				

•			***F*** *amilial*	FBC/FBOC
	•	•	***S*** *trongly* *** S*** *uspected ****F*** *amilial*	**SFBC+ **SFBOC+
Male BC, regardless of family history			***M*** *ale ****B*** *reast ****C*** *ancer*	MBC

**Slightly increased risk**				

	•		***S*** *uspected ****F*** *amilial*	**SFBC/**SFBOC
		•	***S*** *uspected ****F*** *amilial*	**SFBC/**SFBOC
BC/OC without any of the described criteria			***S*** *poradic ****B*** *reast ****C*** *ancer*	SpBC/SpOC

° male relatives excluded when calculating the degree of relationship

* If at least two of the malignancies are OC, the pedigree must be classified as HBOC even if the third criterion is not fulfilled.

**At least two cancer cases are required

HBC – hereditary breast cancer; HBOC -hereditary breast/ovarian cancer; SHBC-suspected hereditary breast cancer; SHBOC-suspected hereditary breast/ovarian cancer; EOBC-early onset breast cancer; BOC-breast ovarian cancer; FBC-familial breast cancer; FBOC-familial breast/ovarian cancer; SFBC+- strongly suspected familial breast cancer; SFBOC+- strongly suspected familial breast/ovarian cancer; MBC-male breast cancer; SFBC- weakly suspected familial breast cancer; SFBOC- weakly suspected familial breast/ovarian cancer; SpBC- sporadic breast cancer; SpOC-sporadic ovarian cancer

**Table 2:** Patient characteristics

**Risk group**	Life time risk of BC* (%)	**N° of women**	**Mean age at surveillance Years (Range)**	**N° with 1**^st^**/2**^nd^**degree relatives**
Carriers	50–80	48	42 (20–75)	40/8
High	30–50	674	42 (15–75)	500/174
Intermediate	18–30	257	43 (19–67)	229/28
Slightly increased	6–18	346	40 (18–75)	303/43

BC – breast cancer

*According to Gail model

**Table 3:** Screening program for each risk category

**Risk Categories**	**Age at the beginning**	**Interval between CBE and US**	**Interval between mammography**	**Interval between MRI**	**Interval between transvaginal US and Ca.125 serum levels**
BRCA+	25	6 months	Annually	Annually	6 months
High risk	30	6 months	Every 2 years until age 36, then annually		Annually
Intermediate risk	30	6 months	2 years until 40, then annually		Annually
Slightly increased risk	30	Annually	One before 40 years, then every 18–24 months		

CBE – clinical breast exam; US – ultrasound; MRI – magnetic resonance imaging.

**Table 4:** Characteristics of patients with interval cancer

**Age (years)**	**Risk group**	**Detection method**	**Time from the last negative exam (months)**	**Type of tumor**	**Tumor size (cm)**	**Stage**	**Grading**	**Hormonal receptors ER/PgR (%)**	**Ki67 (%)**
49	Intermediate	CBE	10	Inflammatory	4*	IV	3	0/0	nd
64	Slightly increased	CBE	1	DCI	0.2°	II	3	90/90	10
51	High	CBE	9§	LCI	1.5	I	3	80/80	20
45	Intermediate	CBE	14	DCIS∞	0.6	is	1	0/0	nd
32•	Slightly increased	CBE+US	10	LCI	2	III	3	80/30	20
47	BRCA+	CBE+US	3^®^	DCI	1.2	I	3	0/0	60
45	High	CBE+US^©^	5	DCI	0.7	I	3	70/80	60
46	Intermediate	CBE+US+Mx^©^	10	DCI	1.6	II	3	0/0	10

* The tumor size was determined at the time of mastectomy, after 8 cycles of preoperative chemotherapy

° Patient was diagnosed with axillary node involvement in the absence of radiological mammary signs; at the left upper outer quadrantectomy, a 0.2 cm focus of DCI was evidenced

§Nine months prior to diagnosis, a fine needle aspiration of microcalcification clusters was negative for atypical cells

∞ Eleven months prior to diagnosis, a nipple discharge cytology was negative for atypical cells. Then she spontaneously contacted a surgeon who performed a subareolar biopsy that showed multiple foci of DCIS. She subsequently underwent mastectomy.

• No mammography was done before the diagnosis in consideration of the slightly increased risk and the young age of the patient

^®^Three months prior to diagnosis, an MRI showed a 9 mm lump in the lower left outer quadrant, with a fast wash in and wash out which was diagnosed as an intramammary lymph node.

^© ^All preceding mammograms were retrospectively evaluated and were negative for suspicious signs.

Note.nd -not determined ;ER – estrogen receptor; PgR – progesterone receptor; DCI -ductal carcinoma infiltrating ; LCI – lobular carcinoma infiltrating; DCIS – ductal carcinoma in situ

**Table 5:** Diagnostic sensitivities for the different imaging modalities for the 36 screen-detected BC

	**Mx**		**US**		**Mx+US**		MRI*	
	Sensitivity**		Sensitivity**		Sensitivity**		Sensitivity**	
	(%)	TP/TP+FN	(%)	TP/TP+FN	(%)	TP/TP+FN	(%)	TP/TP+FN
**All women**	78	28/36	50	18/36	97	35/36	100	4/4

**< 50 years**	64	7/11	64	7/11	100	11/11		
≥ **50 years**	84	21/25	44	11/25	96	24/25	100	4/4

**BRCA+**	50	2/4	75	3/4	75	3/4	100	4/4
**High risk**	90	19/21	52	11/21	100	21/21		
**Intermediate risk**	50	4/8	50	4/8	100	8/8		
**Slightly increased risk**	100	3/3	0	0/3	100	3/3		

Note.-  Mx = Mammography; US = Ultrasonography; MRI = Magnetic resonance imaging

* MRI was performed only in women who were BRCA carriers

** Sensitivity was defined as the percentage of cancers detected (with a specific modality) among all cancers detected with any modality: TP/(TP+FN) where TP is true-positive and FN is false-negative

**Table 6:** Observed and Expected Numbers of Breast Cancer per Risk Group

Risk group	N° of women	Observed n° of breast cancer	N°of person-years at risk	Detection rate per 1000 (95%CI)	Expected N° of breast cancer^§^	Ratio of Observed to Expected (SIR)^®^	*p*
BRCA1/2	48	5	158	31.6 (3.7–53.5)	0.13	2320.3(3.1–83.9)	<0.001
High	674	23	3356	6.9 (1.9–10.6)	5.18	3 4.5 (1.5–8.3)	<0.001
Intermediate	257	11	1108	9.9 (1.3–13.5)	1.23	7.0 (2.0–17.1)	0.0018
Slightly increased	346	5	1444	3.5 (0.1–6.0)	1.17	2.4 (0.9–8.3)	0.76
Overall	1325	44	6066	7.3 (2.2–11.1)	9.03	4.9 (1.6–7.6)	<0.001

§For age-matched population according to Modena Cancer Registry 1998-2002.

^®^• SIR = Standard Incidence Ratio
